# Neuroprotective Effects of Phlorotannin-Rich Extract from Brown Seaweed *Ecklonia cava* on Neuronal PC-12 and SH-SY5Y Cells with Oxidative Stress

**DOI:** 10.4014/jmb.1910.10068

**Published:** 2019-11-22

**Authors:** Jin Ah Nho, Yong Sub Shin, Ha-Ram Jeong, Suengmok Cho, Ho Jin Heo, Gun Hee Kim, Dae-Ok Kim

**Affiliations:** 1 Department of Food Science and Biotechnology, Kyung Hee University, Yongin 704, Republic of Korea; 2 Graduate School of Biotechnology, Kyung Hee University, Yongin 17104, Republic of Korea; 3 Department of Food Science and Technology, Pukyong National University, Busan 4851, Republic of Korea; 4 Division of Applied Life Science (BK21 Plus), Institute of Agricultural and Life Science, Gyeongsang National University, Jinju 52828, Republic of Korea; 5 Department of Foods and Nutrition, Duksung Women’s University, Seoul 01369, Republic of Korea

**Keywords:** Cholinesterase, dieckol, neuron, neuroprotection, vitamin C equivalent antioxidant capacity

## Abstract

Neurodegenerative disorders in the elderly are characterized by gradual loss of memory and cognitive function. Oxidative stress caused by reactive oxygen species is associated with progressive neuronal cell damage and death in Alzheimer’s disease, one of the most common neurodegenerative disorders. An edible brown seaweed, *Ecklonia cava*, contains a variety of biologically active compounds such as phlorotannins. In this study, we comparatively evaluated the total phenolic content, antioxidant capacity, and neuroprotective effects of the phlorotannin-rich extract from *E. cava* (PEEC). The total phenolic content of PEEC and dieckol was 810.8 mg gallic acid equivalents (GAE)/g and 996.6 mg GAE/g, respectively. Antioxidant capacity of PEEC was 1,233.8 mg vitamin C equivalents (VCE)/g and 392.1 mg VCE/g determined using ABTS and DPPH assays, respectively, while those of dieckol were 2,238.4 mg VCE/g and 817.7 mg VCE/g. High-performance liquid chromatography results revealed 48.08 ± 0.67 mg dieckol/g of PEEC. PEEC had neuroprotective effects in pheochromocytoma (PC-12) and human neuroblastoma (SH-SY5Y) cells against H_2_O_2_- and AAPH-induced oxidative damage, partly due to reduced intracellular oxidative stress. PEEC treatment inhibited acetylcholinesterase and butyrylcholinesterase in a dose-dependent manner. Taken together, these findings suggest that PEEC is a good source of antioxidants and neuroprotective materials.

## Introduction

Alzheimer’s disease (AD) is a neurodegenerative disease of the elderly characterized by memory deficits and cognitive impairments due to progressive degeneration and/or death of neurons in the brain [1, 2]. As the average life span increases, so does the number of people with dementia. According to World Health Organization statistics [3], the total number of people with dementia is estimated to increase to 82 million by 2030 and 152 million by 2050. Drugs such as donepezil, galantamine, memantine, and rivastigmine may slow progress of AD but are not cures. Furthermore, usage of these drugs is limited due to side effects such as nausea and vomiting. Thus, continuous research and discovery of natural drugs for prevention and treatment of AD are necessary.

Oxidative stress, an imbalance between the generation and elimination of reactive oxygen species (ROS), produces an excessive amount of free radicals and oxidants in the body [4]. Excessive production of ROS, such as hydroxyl radicals, hydrogen peroxide, superoxide, and hypochlorous acid, causes oxidative damage to cellular macromolecules such as DNA, proteins, and lipids and impairs mitochondrial function [5]. Oxidative stress is associated with neuronal cell damage and death in neurodegenerative diseases such as AD, Huntington’s disease, and Parkinson’s disease [6]. The brain is particularly vulnerable to oxidative stress due to high levels of polyunsaturated fatty acids, low levels of antioxidants, and high oxygen consumption [7]. Excess exposure of the brain to oxidative stress can damage neuronal structures, leading to loss of neurons. Strategically, dietary antioxidants are recommended for prevention and/or delay of neuro-degeneration caused by oxidative stress [8]. The neurotransmitter acetylcholine is hydrolyzed to choline and acetate by acetylcholinesterase (AChE), terminating acetylcholine-mediated neurotransmission [9]. Butyryl-cholinesterase (BChE) is a hydrolase found in cholinergic neurons in the nervous system of the human brain and also hydrolyzes acetylcholine to choline and acetate [10]. Levels of AChE and BChE in the brain are increased in Alzheimer’s disease [10]. Therefore, reversible inhibitors of AChE and BChE can potentially maintain the levels of the neurotransmitter acetylcholine by inhibiting their activities in AD patients. Research on various natural antioxidants, including phenolics and vitamins, has mainly focused on terrestrial plants such as herbs, fruits, and vegetables [11]. However, relatively limited information is available about the neuroprotective effects of natural antioxidants and cholinesterase inhibitors derived from marine resources.

*Ecklonia cava* (EC), an edible brown seaweed, is distributed on the southern coast of the Korean Peninsula. EC contains a variety of bioactive compounds, such as phlorotannins, peptides, carotenoids, and fucoidans [12, 13]. Phlorotannins, a type of tannin found in brown algae, are polymers composed of a phloroglucinol (1,3,5-trihydroxybenzene) monomer unit with varying degrees of polymerization and bonding [14]. Phlorotannins from EC formed as secondary phenolic metabolites include dieckol, phlorofucofuroeckol, 6,6’-bieckol, and eckol [15]. Phlorotannins have been reported to have a variety of beneficial properties, including antioxidant, anti-inflammatory, anti-allergic [16], anti-diabetic [17], hypnotic [18], and neuroprotective effects [19, 20].

Studies have reported diverse neuroprotective effects of marine algae EC [19, 20], but limited research has assessed the neuroprotective effects of phlorotannins in EC. In this study, we investigate total phenolic content, antioxidant capacity, and neuroprotective effects of phlorotannin-rich extract from EC (PEEC) and dieckol against oxidative stress in rat pheochromocytoma (PC-12) and human neuroblastoma (SH-SY5Y) cells. Anticholinesterase activity of PEEC and dieckol was also examined using AChE and BChE inhibition assays. High-performance liquid chro-matography (HPLC) was used to quantify dieckol, a phlorotannin in PEEC.

## Materials and Methods

### Chemicals

Acetylthiocholine iodide (ATCI), AChE, ascorbic acid, 2,2’-azino-bis(3-ethylbenzothiazoline-6-sulfonic acid) diammonium salt (ABTS), 2,2’-azobis(2-methylpropionamidine) dihydrochloride (AAPH), BChE, S-butyrylthiocholine chloride (BTCC), 2’,7’-dichlorofluorescin diacetate (DCFH-DA), dimethyl sulfoxide (DMSO), 3-(4,5-dimethyl-2-thiazolyl)-2,5-diphenyl-2H-tetrazolium bromide (MTT), 2,2-diphenyl-1-picrylhydrazyl (DPPH), 5,5’-dithiobis(2-nitrobenzoic acid) (DTNB), Folin-Ciocalteu’s phenol reagent, gallic acid, hydrogen peroxide, and phosphate-buffered saline (PBS) were purchased from Sigma-Aldrich Co., LLC (USA). Dulbecco’s phosphate-buffered saline (DPBS), fetal bovine serum (FBS), Hanks’ balanced salt solution (HBSS), Minimum Essential Medium Eagle (MEM), penicillin-streptomycin solutions, Roswell Park Memorial Institute (RPMI)-1640, and trypsin-EDTA were purchased from Welgene Inc. (Korea). Dieckol, a standard compound isolated from the phlorotannin-rich extract using silica gel and Sephadex LH-20 column chromatography [21], was obtained from S&D Co., Ltd. (Korea). All reagents were of analytical or HPLC grade unless otherwise specified.

### PEEC Preparation

PEEC was supplied by Seojin Biotech Co., Ltd. (Republic of Korea). EC was collected from Aewol-eup, Jeju-island, Republic of Korea. Fresh EC was washed with distilled water and dried. Dry EC was extracted with 10 multiples of 50% (v/v) fermentation ethanol at 60°C for 6 h. The extract was filtered through Whatman no. 2 filter paper (Whatman International Ltd., England), concentrated, and then lyophilized.

### Determination of Total Phenolic Content

Total phenolic content was measured with a colorimetric assay using Folin-Ciocalteu’s phenol reagent [22]. An aliquot (0.2 ml) of appropriately diluted PEEC or dieckol was mixed with 2.6 ml of deionized water. At 0 min, 0.2 ml of Folin-Ciocalteu’s phenol reagent was added to the mixture. At 6 min, 2 ml of 7% (w/v) Na_2_CO_3_ solution was added. At 90 min, absorbance was measured at 750 nm using a SPECTRONIC 200 spectrophotometer (Thermo Fisher Scientific Inc., USA). Total phenolic content was expressed as mg gallic acid equivalents (GAE)/g.

### Determination of Antioxidant Capacity

Antioxidant capacity was measured using ABTS and DPPH radicals and expressed as mg vitamin C equivalents (VCE)/g. In the ABTS radical scavenging assay [23], ABTS radical solution was adjusted to an absorbance of 0.650 ± 0.020 at 734 nm. The reaction between ABTS radicals and appropriately diluted PEEC or dieckol was allowed to proceed at 37oC for 10 min, and the absorbance of the resulting solution was measured at 734 nm using a SPECTRONIC 200 spectrophotometer (Thermo Fisher Scientific Inc.).

In the DPPH radical scavenging assay [24], DPPH radicals (0.1 mM) were dissolved in 80% (v/v) aqueous methanol. Absorbance of DPPH radicals was set to 0.650 ± 0.020 at 517 nm. The reaction between DPPH radicals and the appropriately diluted PEEC or dieckol was allowed to proceed at 23°C for 30 min. The absorbance of the resulting solution was monitored at 517 nm using a SPECTRONIC 200 spectrophotometer (Thermo Fisher Scientific Inc.).

### Quantification of Dieckol in PEEC using Reversed-Phase HPLC System

The amount of dieckol in PEEC was analyzed using a reversed-phase HPLC system (Agilent 1200; Agilent Technologies, Inc., USA) equipped with quaternary pump, autosampler, vacuum degasser, thermostatted column compartment, and diode array detector. Chromatographic separation was performed using a C18 reversed-phase analytical column (Kromasil 100-5-C18, 4.6 × 250 mm, 5 μm; Eka Chemicals AB, Sweden) with injection volume of 5 μl. The flow rate was maintained at 0.7 ml/min. The detection wavelength was 230 nm. The gradient of two mobile phases, deionized water (solvent A) and absolute methanol (solvent B), was as follows; 80% A/20% B at 0 min, 60% A/40% B at 10 min, 40% A/60% B at 40 min, 20% A/80% B at 45 min, 80% A/20% B at 55 min, and 80% A/20% B at 60 min. Dieckol in PEEC was quantified using the standard curve of authentic standard dieckol.

### Cell Culture

Two neuronal cell lines (PC-12 and SH-SY5Y) were used to evaluate the neuroprotective effects of PEEC and dieckol against oxidative stress in vitro. The PC-12 cell line is derived from a transplantable rat pheochromocytoma and was purchased from American Type Culture Collection (USA). PC-12 cells were cultured in RPMI-1640 medium containing 10% heat-inactivated FBS, 100 units/ml penicillin, and 100 μg/ml streptomycin. The human neuroblastoma SH-SY5Y cell line was obtained from the Korean Cell Line Bank (Korea). SH-SY5Y cells were cultured in MEM containing 10% heat-inactivated FBS, 100 units/ml penicillin, and 100 μg/ml streptomycin. Both neuronal cell lines were cultured in a humidified incubator (CO_2_ incubator BB 15; Thermo Electron LED GmbH, Germany) with 5% CO_2_ at 37°C.

### Cytotoxicity and Cell Viability

The effects of PEEC and dieckol on the cytotoxicity and cell viability of both PC-12 and SH-SY5Y cell lines were estimated using MTT reduction assay [25]. PC-12 cells at a density of 2 × 104 cells/well in 96-well plates were pre-cultured for 24 h. After removal of the medium, PC-12 cells were treated with serum-free medium containing various concentrations of PEEC and dieckol for 24 h. PC-12 cells were treated with 300 μM H_2_O_2_ for 1 h and then with 0.5 mg/ml MTT for 3 h. Conversely, SH-SY5Y cells in 96-well plates at a density of 1 × 10^5^ cells/well were pre-cultured for 24 h. After removal of the medium, SH-SY5Y cells were treated with serum-free medium containing various concentrations of PEEC and dieckol for 24 h. After 24 h, SH-SY5Y cells were treated with 100 μM H_2_O_2_ for 1 h, and then with 0.5 mg/ml MTT for 3 h. The resulting formazan products from both cell lines were dissolved by addition of DMSO. The amount of MTT formazan dissolved in DMSO was determined by measuring absorbance with a microplate reader (Infinite M200; Tescan Austria GmbH) at 570 nm (detection wavelength) and 630 nm (reference wavelength). The cytotoxicity and viability of both cell lines were expressed as percentage (%) of viable cells relative to control cells (100%).

### Determination of Intracellular Oxidative Stress Level

Intracellular oxidative stress level was evaluated using the fluorescent probe DCFH-DA [26]. PC-12 (2 × 10^4^ cells/well) and SH-SY5Y (1 × 10^5^ cells/well) cells were pre-cultured for 24 h. This was followed by treatment with various concentrations of PEEC and dieckol for 24 h. After removing the supernatant, both cell lines were incubated with 50 μM of DCFH-DA in HBSS f or 1 h. PC-12 cells were separately treated with 30 μM of AAPH and 100 μM of H_2_O_2_ in HBSS forhile SH-SY5Y cells were separately treated with 10 μM of AAPH and 100 μM of H_2_O_2_ in HBSS for 1 h. Fluorescence was measured at 485 nm (detection wavelength) and 535 nm (emission wavelength) using a microplate reader (Infinite M200; Tecan Austria GmbH). The intracellular oxidative stress level was expressed as percentage (%) decrease in fluorescence intensity compared to the control (100%).

### Inhibitory Effects on Cholinesterases

Inhibitory effects of PEEC and dieckol on cholinesterases (AChE and BChE) were assessed by a modified method of Ellman *et al*. [27]. To evaluate AChE inhibition, 20 μl of PEEC or dieckol at various concentrations was mixed with 150 μl of DPBS. At zero min, 20 μl of 15 mM ATCI and 30 μl of 10 mM DTNB were added to the mixture. After incubation at 37°C for 10 min, 20 μl of AChE (0.2 U/ml) was added. After reaction at 37°C for 30 min, absorbance was measured at 415 nm using a microplate reader (Infinite M200; Tecan Austria GmbH). BChE inhibition was evaluated by adding 20 μl of BTCC (10 mM) and 20 μl of BChE (0.06 U/ml) instead of ATCI and AChE. The percentage (%) inhibition of AChE and BChE was calculated using the equation

$$\mathrm{Percentage}(\%)\mathrm{inhibition}=([A_{\mathrm{control}}–A_{\mathrm{sample}}/A_{\mathrm{control}})\times 100$$

where *A*_control_ is absorbance of the control at 30 min, and *A*_sample_ is absorbance of the sample at 30 min.

### Statistical Analysis

Data are expressed as mean ± standard deviation of three replicate determinations. One-way analysis of variance was applied to determine the significance of differences among means. Statistical analyses were conducted using Duncan’s multiple range test using SAS software (version 9.4; SAS Institute Inc., USA) with significance set to *p* < 0.05.

## Results

### Total Phenolic Content and Antioxidant Capacity

Total phenolic content and antioxidant capacity of PEEC and dieckol are shown in Table 1. The total phenolic content of PEEC and dieckol was 810.8 mg GAE/g and 996.6 mg GAE/g, respectively. Antioxidant capacity of PEEC was 1,233.8 mg VCE/g and 392.1 mg VCE/g as determined using ABTS and DPPH assays, respectively. Dieckol showed antioxidant capacity of 2,238.4 mg VCE/g with ABTS assay and 817.7 mg VCE/g with DPPH assay.

### HPLC Analysis

**Fig. 1:**
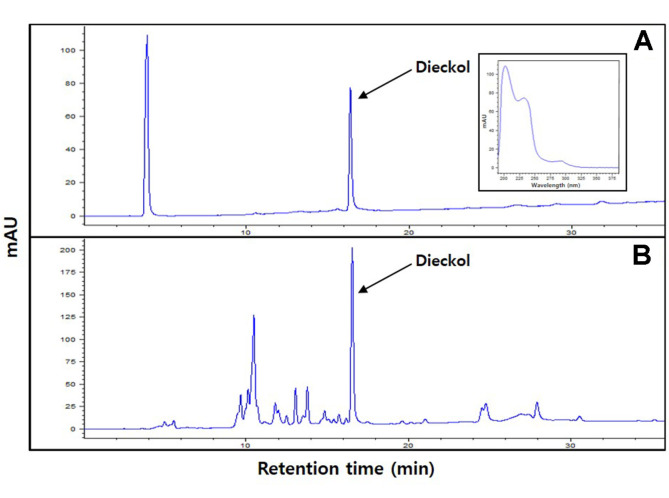
HPLC traces of the (**A**) dieckol standard and (**B**) phlorotannin-rich extract from *Ecklonia cava* at 230 nm.

Using reversed-phase HPLC analysis, dieckol eluted at a 16.521 min retention time (Fig. 1). PEEC contained 48.08 ± 0.67 mg dieckol/g (data not shown).

### Neuroprotective Effects of PEEC and Dieckol against Oxidative Stress in Neurons

**Fig. 2:**
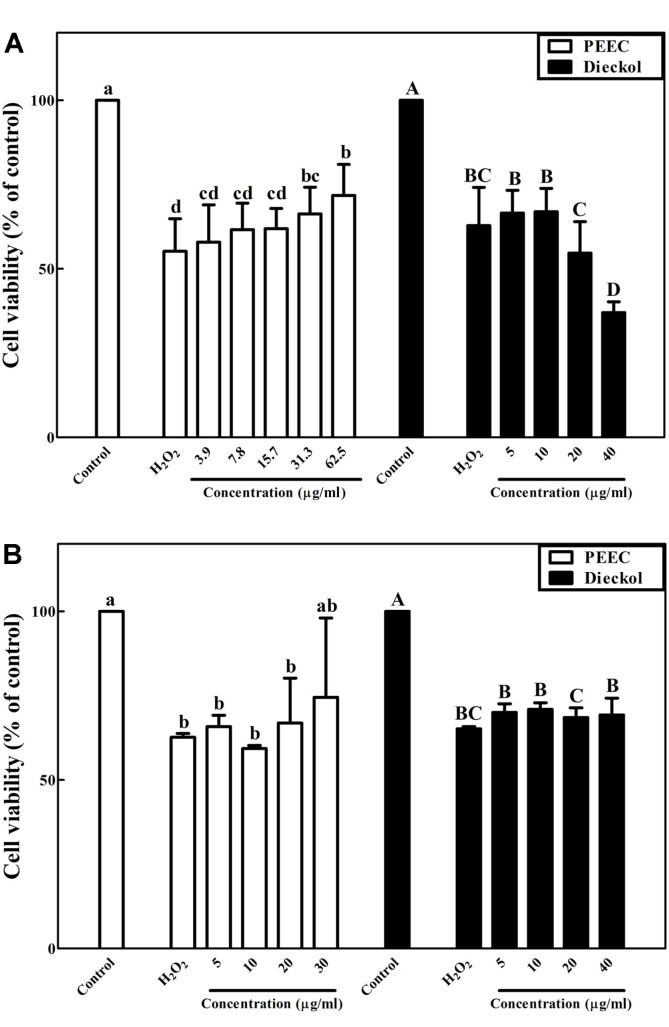
Neuroprotective effects of the phlorotannin-rich extract from *Ecklonia cava* (PEEC) and dieckol on neuronal (**A**) PC-12 and (**B**) SH-SY5Y cells against oxidative stress induced with hydrogen peroxide (H_2_O_2_) using the MTT assay. Data are displayed as mean ± standard deviation (bars) of three replicates. Different lowercase and uppercase characters on bars in PEEC and dieckol treatments indicate significant differences according to Duncan’s multiple range test (*p* < 0.05).

The cytotoxicity of PEEC and dieckol was examined at various concentrations. Cell viability of 90% or above was considered lack of cell cytotoxicity. PEEC and dieckol had no cytotoxicity against PC-12 cells up to 62.5 μg/ml and 40.0 μg/ml, respectively (data not shown). Treatment with hydrogen peroxide at 300 μM resulted in approximately 38.2–44.8% decrease of cell viability in PC-12 cells compared with the control (100%) (Fig. 2A). Pretreatment of PC-12 cells with PEEC increased the viability of PC-12 cells exposed to oxidative stress compared with the stress control (Fig. 2A). Treatment with PEEC of 62.5 μg/ml significantly (*p* < 0.05) increased PC-12 cell viability up to about 71.8% compared with the stress control (55.2%)(Fig. 2A). However, treatment with dieckol had no significant (*p* > 0.05) effect compared with the stress control (62.8%) (Fig. 2A).

Both PEEC and dieckol had no cytotoxicity against dopaminergic neuronal SH-SY5Y cells up to 30.0 μg/ml and 40.0 μg/ml, respectively (data not shown). Oxidative stress (100 μM H_2_O_2_) reduced the viability of SH-SY5Y cells to approximately 62.7–65.2% compared with the control (100%) (Fig. 2B). Pretreatment of cells with PEEC and dieckol resulted in no significant (*p* > 0.05) increase in viability of SH-SY5Y cells exposed to oxidative stress compared with the stress control (Fig. 2B).

### Intracellular Oxidative Stress 

**Fig. 3:**
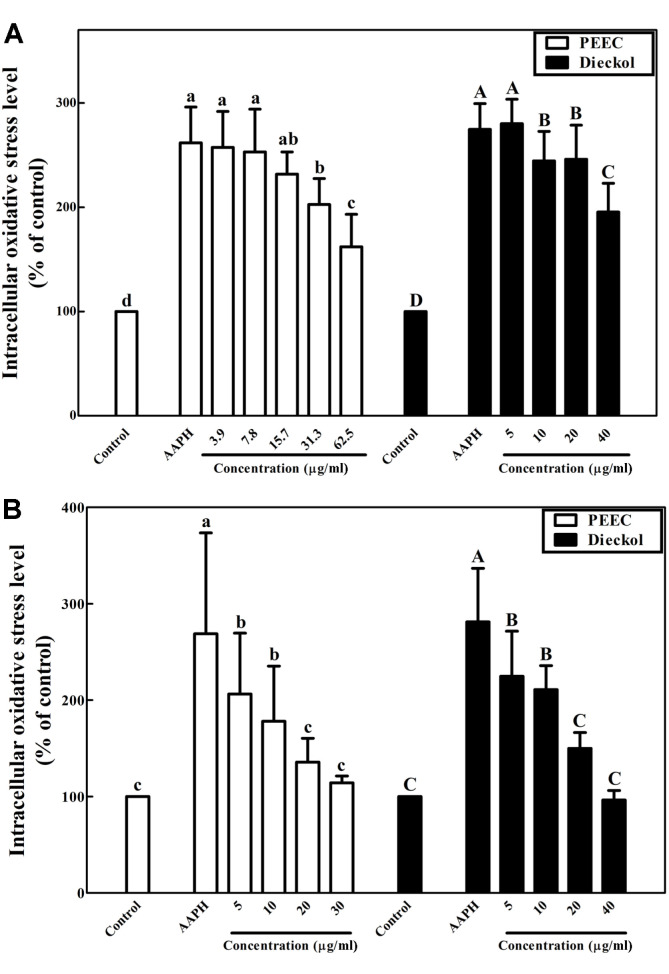
Effects of the phlorotannin-rich extract from *Ecklonia cava* (PEEC) and dieckol on intracellular oxidative stress in neuronal (**A**) PC-12 and (**B**) SH-SY5Y cells against oxidative stress induced with AAPH using the DCFH-DA assay. Data are displayed as mean ± standard deviation (bars) of three replicates. Different lowercase and uppercase characters on bars in PEEC and dieckol treatments indicate significant differences according to Duncan’s multiple range test (*p* < 0.05).

We evaluated whether PEEC and dieckol reduce AAPH-induced oxidative stress in PC-12 and SH-SY5Y cells (Fig. 3). As shown in Fig. 3A, oxidative stress (261.8%) following only AAPH treatment was attenuated to 162.1% after treatment with 62.5 μg/ml PEEC. Treatment with 40.0 μg/ml dieckol decreased oxidative stress to 195.4% compared to the stress control (274.7%) treated with AAPH only. Exposure of SH-SY5Y cells to 10 μM of AAPH increased intracellular oxidative stress level to approximately 270% (Fig. 3B). Treatment with 30.0 μg/ml PEEC and 40.0 μg/ml dieckol attenuated intracellular oxidative stress to 114.2% and 96.4% in SH-SY5Y cells, respectively (Fig. 3B).

**Fig. 4:**
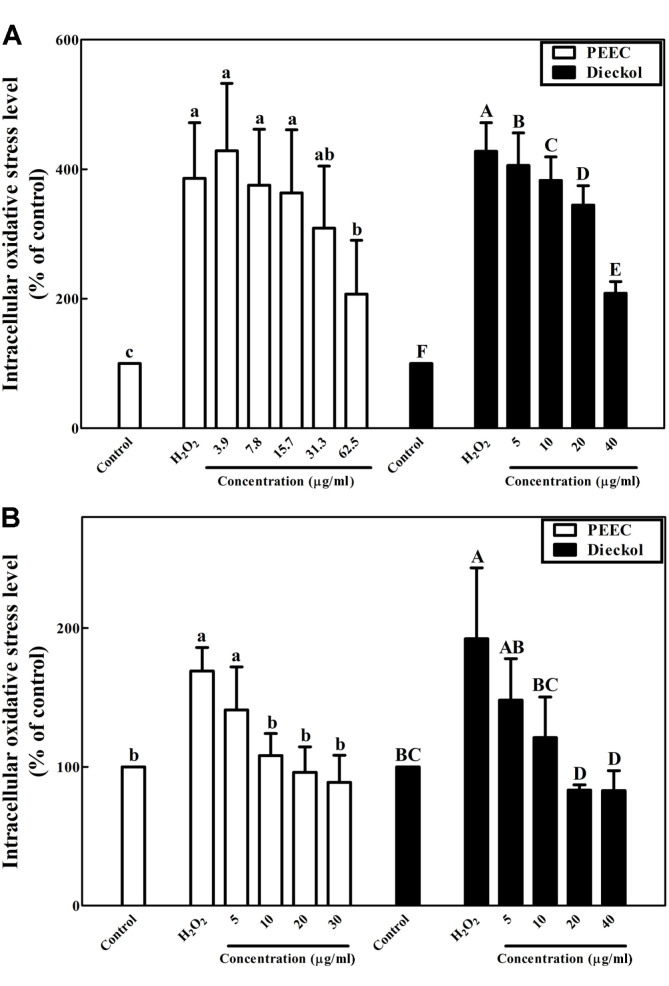
Effects of the phlorotannin-rich extract from *Ecklonia cava* (PEEC) and dieckol on intracellular oxidative stress in neuronal (**A**) PC-12 and (**B**) SH-SY5Y cells against oxidative stress induced with hydrogen peroxide (H_2_O_2_) using the DCFH-DA assay. Data are displayed as mean ± standard deviation (bars) of three replicates. Different lowercase and uppercase characters on bars in PEEC and dieckol treatments indicate significant differences according to Duncan’s multiple range test (*p* < 0.05).

We also evaluated whether PEEC and dieckol scavenge H_2_O_2_-induced oxidative stress in PC-12 and SH-SY5Y cells (Fig. 4). Exposure of PC-12 cells to 100 μM H_2_O_2_ increased the intracellular oxidative stress level up to 386.0% and 427.7% in treatments with PEEC and dieckol, respectively, whereas treatments with 62.5 μg/ml PEEC and 40.0 μg/ml dieckol attenuated intracellular oxidative stress to 207.1% and 208.1% (Fig. 4A). Exposure of SH-SY5Y cells to 100 μM H_2_O_2_ showed intracellular oxidative stress levels of 169.1% and 192.3% in treatments with PEEC and dieckol, respectively, whereas treatments with PEEC (30.0 μg/ml) and dieckol (40.0 μg/ml) decreased intracellular oxidative stress to 88.9% and 82.9% (Fig. 4B).

### Cholinesterase Inhibitory Activity of PEEC and Dieckol

**Fig. 5:**
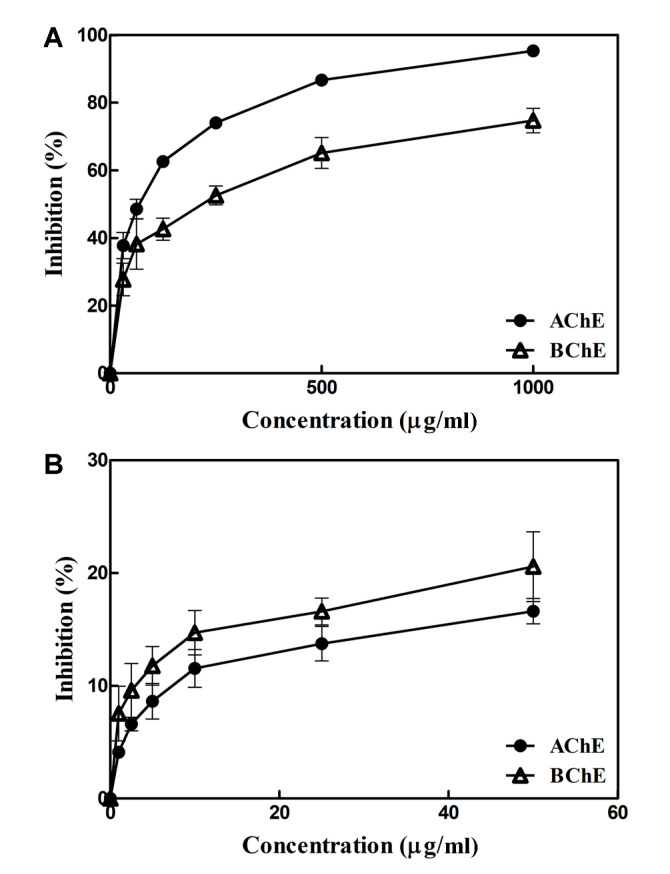
Inhibitory effects of the (**A**) phlorotannin-rich extract from *Ecklonia cava* and (**B**) dieckol on acetylcholinesterase (AChE) and butyrylcholinesterase (BChE) activities.

Figure 5A shows that PEEC at various concentrations inhibited the activity of cholinesterases (AChE and BChE). At 1,000 μg/ml of PEEC, AChE and BChE inhibition was approximately 95.4% and 74.7%, respectively, while those at 31.25 μg/ml of PEEC were approximately 37.8% and 27.8%. The IC_50_ values of PEEC on inhibition of AChE and BChE were estimated as 68.9 μg/ml and 217.7 μg/ml, respectively.

As shown in Fig. 5B, dieckol at various concentrations inhibited the activity of cholinesterases (AChE and BChE). Unlike the results of PEEC inhibitory effects, dieckol showed lower inhibitory effect on AChE than BChE. At 50.0 μg/ml of dieckol, AChE and BChE inhibitions were approximately 16.6% and 20.6%, respectively. The IC_50_ values of dieckol on inhibition of AChE and BChE could not be determined in the concentration ranges used in this study (Fig. 5B).

## Discussion

The PEEC used in this study was found to contain a large amount of total phenolics (Table 1). EC was reported to show the highest total phenolic content among brown seaweeds [28]. The total phenolic content (810.8 mg GAE/g) of PEEC was about 9.1 times higher than that of the aqueous ethanolic EC extract previously reported by Lee *et al*. [13] and about 9.8 times higher than that of the aqueous methanolic EC extract previously reported by Senevirathne *et al*. [29]. PEEC was extracted using cyclic extraction and continuous centrifugation with high solvent temperature in this study. The higher total phenolic content of PEEC is partly due to different harvest times and extraction methods of EC compared to other studies [13].

Marine algae off the coast of Korea have been reported to show strong antioxidant capacity [30]. EC has been found to have the highest antioxidant capacity among 10 different marine algae [31]. One gram of PEEC used in this study had approximately 23% higher and 61% lower antioxidant capacity compared with the same weight of vitamin C by ABTS and DPPH assays, respectively (Table 1). This discrepancy of PEEC antioxidant capacity may be due in part to the use of different radicals and reaction media in each assay [32, 33]. EC has been reported to contain various phlorotannins, including 7-phloroeckol, 6,6’-bieckol, eckol, fucodiphloroethol G, phlorofucofuroeckol A, phloroglucinol, and dieckol [34]. Phlorotannins from marine algae were previously reported to act as antioxidants for scavenging free radicals [19, 35, 36].

In addition, we investigated whether PEEC and its component dieckol have neuroprotective effects on neuronal PC-12 and SH-SY5Y cells against oxidative stress caused by hydrogen peroxide. Upon exposure to hydrogen peroxide in PC-12 cells, cell viability was significantly (*p* < 0.05) reduced compared to that of control cells (Fig. 2A). PEEC protected PC-12 and SH-SY5Y cells against oxidative damage (Fig. 2). Pretreatment with PEEC at 62.5 μg/ml significantly (*p* < 0.05) increased PC-12 cell viability by about 30% compared with cells under oxidative stress only (Fig. 2A). Pretreatment with PEEC at 30.0 μg/ml increased SH-SY5Y cell viability by approximately 19% compared with cells under oxidative stress only (Fig. 2B). EC extract with antioxidants was previously reported to reduce production and fibrilization of amyloid beta peptide [37, 38]. Phloroglucinol, a building block of phlorotannins such as dieckol, was previously reported to have neuroprotective effects against oxidative stress in SH-SY5Y cells [39]. Phlorofucofuroeckol has been shown to protect PC-12 cells from neurotoxicity induced by glutamate [40]. Dieckol was reported to protect SH-SY5Y cells from oxidative stress and inhibit α-synuclein aggregation in SH-SY5Y cells [41]. However, dieckol had no neuroprotective effect in either cell line (Fig. 2). The viability of PC-12 cells treated with dieckol at 40.0 μg/ml was significantly (*p* < 0.05) reduced compared with that of the stress control (Fig. 2A), indicating that higher concentration of dieckol and hydrogen peroxide promote neuronal cell death partly due to pro-oxidant properties.

An increase in intracellular ROS level leads to apoptosis; therefore, removal of ROS using antioxidants such as phlorotannins has neuroprotective effects against oxidative stress. Two sources of oxidative stress, AAPH and H_2_O_2_, increase intracellular oxidative stress in neuronal cells, such as PC-12 and SH-SY5Y cells [42]. Pretreatment of neuronal PC-12 and SH-SY5Y cells with PEEC and dieckol against oxidative stress induced with hydrogen peroxide and AAPH-generated peroxyl radicals attenuated oxidative stress levels inside neurons (Figs. 3 and 4). Phlorotannins such as dieckol from EC have previously been reported to protect hippocampal neuronal HT-22 cells against H_2_O_2_-induced oxidative stress, partly due to inhibition of lipid peroxidation and ROS accumulation [43]. Dieckol has been reported to reduce oxidative stress caused by the neurotoxic rotenone in SH-SY5Y cells [41]. In this study, dieckol reduced oxidative stress in neuronal cell lines, PC-12 and SH-SY5Y, similar to the results of previous reports [41, 43].

PEEC had higher inhibitory activity of AChE than BChE (Fig. 5A), while dieckol showed higher BChE inhibition than AChE (Fig. 5B). Differences in AChE and BChE inhibitory activities are attributed to different specific binding characteristics of enzyme to substrates such as a single compound (dieckol) and complex PEEC. At similar concentrations, PEEC inhibited AChE and BChE activities more than dieckol (Fig. 5B). Similar to the results of this study, ethanolic extract from EC was reported to exhibit higher AChE inhibitory activity than BChE [44]. Dieckol was previously reported to improve cognitive impairment in ethanol-fed mice, partly due to increased level of acetylcholine and inhibited AChE activity in the brain [45]. It was previously reported that IC_50_ values of dieckol on AChE and BChE inhibition were about 17.1 μM and >500 μM, respectively [44]. In this study, IC_50_ values of dieckol on AChE and BChE inhibition could not be determined because the concentration used in the enzyme assay was much lower than previously reported IC_50_ values of AChE and BChE inhibition [44].

In conclusion, PEEC was determined to contain dieckol, a phlorotannin found in seaweed. PEEC had large amounts of total phenolics and antioxidants. PEEC and dieckol protected neuronal PC-12 and SH-SY5Y cells from intracellular oxidative stress, in part due to their antioxidant capacities. Activities of AChE and BChE were inhibited by PEEC and dieckol, indicating that neurotransmission of acetylcholine can be prolonged in brain neurons. Although dieckol, an indicator component of PEEC, did not significantly increase the neuronal cell viability, other bioactive compounds in PEEC may lead to increased viability in neuronal cells. Our results suggest that PEEC rich in antioxidants can potentially protect neurons from oxidative damage and inhibit hydrolytic activities of cholinesterases. Further research is needed to determine the efficacy in a neurodegenerative animal model in vivo.

## Figures and Tables

**Table 1:** Total phenolic content and antioxidant capacity of the phlorotannin-rich extract from *Ecklonia cava* (PEEC) and dieckol.

	Total phenolic content (mg gallic acid equivalents/g)	Antioxidant capacity (mg vitamin C equivalents/g)	

		ABTS^1^	DPPH^2^
PEEC	810.8 ± 19.4^3^	1,233.8 ± 8.2	392.1 ± 5.8
Dieckol	996.6 ± 34.2	2,238.4 ± 156.1	817.7 ± 39.0

^1^2,2’-Azino-bis(3-ethylbenzothiazoline-6-sulphonic acid) radical scavenging assay

^2^2,2-Diphenyl-1-picrylhydrazyl radical scavenging assay

^3^Data are expressed as mean ± standard deviation (*n* = 3).
